# Pharmacological Management of Esophageal Food Bolus Impaction

**DOI:** 10.1155/2013/924015

**Published:** 2013-05-13

**Authors:** Yasir Mohammed Khayyat

**Affiliations:** Department of Medicine, Umm Al-Qura University, P.O. Box 7607, Makkah, Saudi Arabia

## Abstract

*Background*. Soft esophageal bolus impaction is an emergency that requires skilled endoscopic removal if persistent obstructive symptoms do not resolve spontaneously after careful observation. Expedited care of these patients is crucial to avoid respiratory and mechanical complications. Other possible options for management include medical agents used to manage it prior to performing endoscopy if access to endoscopy was not available or declined by the patient. *Aim*. To review the available pharmacological and other nonmedicinal options and their mechanism of relief for soft esophageal impaction. *Method*. Pubmed, Medline and Ovid were used for search of MESH terms pertinent including “foreign body, esophageal, esophageal bolus and medical” for pharmacological and non medicinial agents used for management of esophageal soft bolus impaction as well as manual review of the cross-references. *Results*. Several agents were identified including Buscopan, Glucagon, nitrates, calcium channel blockers, and papaveretum. Non medicinal agents are water, effervescent agents, and papain. No evidence was found to suggest preference or effectiveness of use of a certain pharmacological agent compared to others. Buscopan, Glucagon, benzodiazepines, and nitrates were studied extensively and may be used in selected patients with caution. Use of papain is obsolete in management of soft bolus impaction.

## 1. Introduction

Foreign body esophageal impaction is a common emergency that ranks third after upper and lower gastrointestinal bleeding. It has annual incidence of 13 : 100 000 among the general population with a male to female predominance 1.7 : 1. The rate of occurrence increases with age, particularly in patients over seventy years [1]. Esophageal impaction can be distinguished into two types: (a) true foreign body impaction caused by objects such as blunt- or sharp-pointed objects and in relation to other miscellaneous objects that could occlude the lumen; (b) food impaction due to nonsolid material in the esophagus [2]. It is managed endoscopically either by pushing or extracting the impacted material in the esophagus using flexible or rigid endoscopy [3–5]. However, a survey conducted among UK practitioners showed that the majority did not usually proceed immediately to rigid esophagoscopy to remove the food bolus impaction mechanically; rather, they prefer to use antispasmodic drugs (83%), the most common being hyoscine butylbromide (Buscopan) and diazepam, to try to induce spontaneous passage of the obstruction [6]. Endoscopic skills to perform upper endoscopy are varied, and any attempt to manage an esophageal impaction is hazardous if a less experienced endoscopist manages unrecognized distal esophageal lesion [7]. Delayed interventions after 24 hours of the symptoms are often associated with longer therapeutic endoscopic time and more symptomatic esophageal ulcerations with odynophagia [8]. This review will outline the available pharmacological agents used for esophageal bolus management and their efficacy.

## 2. Etiology and Risk Factors

Esophageal impaction occurs as a result of a variety of etiologies related to the esophageal mucosal musculature and neuromuscular and luminal pathologies (Box 1); however, the most common obstruction is due to poorly masticated food in edentulous elderly individuals [9]. Certain unusual causes of foreign body impaction have also been noted with herpes simplex and Eosinophilic Esophagitis [10, 11]. Paraesophageal hernia also was reported once and managed conservatively with intravenous Glucagon [12]. The risk factors associated with esophageal impaction include “mental retardation, psychiatric disorders, alcohol ingestion, edentulous elderly individuals, and secondary gain” [13].

## 3. Selected Etiologies

### 3.1. Eosinophilic Esophagitis (EE)

This condition was first recognized in 1995, in children with atopic conditions such as eczema, asthma, and hay fever, and since then had become increasingly recognized in children and adults, among randomly selected Swedish adults (1000 individuals), where a 1% prevalence was detected [10]. Interestingly, this condition showed male predominance (70%) in relation to their fast eating habits. It is found to be a T helper 2 subset-mediated disorder with subsequent IgE sensitization, characterized histologically by dense esophageal eosinophilia, which leads to longstanding inflammation with wall remodeling, thereby making the esophagus fragile and inelastic. Adults frequently present with dysphagia, heartburn [14] as well as chest pain, and esophageal food impaction. The diagnosis is prompted by upper endoscopy which reveals suggestive endoscopic features such as linear furrows, mucosal rings, white papules, or narrowed lumen. Esophageal biopsy is confirmatory of the diagnosis when the eosinophilic infiltrate ≥15 eosinophils per high power field. Several reports showed the presence of EE as an entity in cohorts of patients presenting with food impaction. Remedios et al., reported that 29 of the 43 patients with eosinophilic esophagitis were diagnosed based on biopsy [16]. Endoscopic management of food bolus requires diligence, as reported by Straumann et al., among a Swedish cohort of 251 patients, endoscopy-related perforations occurred in three of them [15]. The management of EE in an acute setting during esophageal impaction is mainly endoscopic based, with gentle manipulation of the bolus using the endoscopic accessories. No report of the use of a pharmacological agent which has a positive effect on disimpaction of the bolus is available. Chronic management with nonpharmacological options, using the elemental diet with elimination of certain food items known to have a propensity to induce allergy, has been found to be successful. The pharmacological agents used are swallowed corticosteroids (fluticasone) which are widely used to topically inhibit the inflammatory process; other agents include systemic corticosteroids, proton pump inhibitor, and the leukotriene receptor antagonist montelukast. Other innovative molecules reported in the treatment of EE are mepolizumab, a monoclonal antibody against interleukin 5 [16]; however, the latter still has limited use in the regular treatment of EE.

## 4. Clinical Features and Investigations

Typically the patient complains of sudden onset of dysphagia during a meal, odynophagia, chest pain, or inability to tolerate secretions (sialorrhea). He/she may be able to identify the material swallowed but cannot clearly localize it. It is usually traced back to meals shared with other family members, most often meat or steak at parties or family gatherings. Interestingly, EE-related food bolus impaction has been noted to occur during the summer and fall months [17], implying that this is a seasonal variation related to exposure to the aeroallergens prevalent during those months of the year. On physical examination, the vital signs at the time of emergency room presentation could show hypoxemia, tachycardia, and high blood pressure, particularly during prolonged periods of bolus obstruction, associated with airway compromise and excessive coughing. Limited physical findings with erythema, tenderness, and crepitus have been noted which could manifest as a result of oropharyngeal or proximal esophageal perforation. Drooling of saliva is suggestive of esophageal obstruction [18, 19]. The diagnosis is usually made based on clinical grounds; however, in suspected cases, the diagnosis of metallic versus soft impaction is confirmed by performing plain single and biplanar views of the neck and chest X-ray. Contrast examination using Barium or Gastrografin is not advocated due to coating of the contrast material which obstructs further endoscopic examination, as well as the anticipated risk of aspiration into the lungs. A CT scan of the neck and chest is not normally required unless the suspicion of perforation is high. Marshmallow pieces in a standard bolus were used with fluoroscopic examination to investigate the cause of dysphagia in nonacute settings such as rings, strictures, and hiatal hernia [20].

## 5. Management Options of Impacted Food Bolus

Endoscopic removal of upper gastrointestinal tract foreign bodies and food bolus impaction has been found to be efficacious and safe, using several methods, particularly the Roth net. Other accessories used are dormia baskets, retrieval forceps, and polypectomy snares [21]. As discussed earlier, due to the difficulties related to endoscopy, pharmacologic agents such as Buscopan, Glucagon, papaveretum, benzodiazepines, calcium channel blocker, and nitrates are the available medical options. Nonpharmacologic agents include Papain, water, and effervescent agents.

### 5.1. Buscopan

This is a peripherally acting antimuscarinic and anticholinergic agent whose antispasmodic activity relaxes the lower esophageal sphincter. Reports of its use are varied in the efficacious management of soft food bolus dislodgement. Basavaraj et al. showed that the dislodgement of the food bolus after IV Buscopan does not correlate with the type of food or the duration of symptomatic relief of impaction, prior to its administration [22]. Other reports by Anderson showed no difference in the spontaneous dislodgment of the food bolus among patients who received IV Buscopan versus those who did not [23]. Such conflicting reports do not strongly support the routine use of the drug, due to the variable responses. It is contraindicated in elderly patients with coexistent glaucoma or prostatism due to its inherited pharmacological properties.

### 5.2. Glucagon

This polypeptide, secreted from the alpha-cells of the islets of Langerhans in the pancreas which was first purified in 1955, has a cardiovascular effect, inducing the relaxation of the smooth muscles of the genitourinary and biliary tree. The gastrointestinal effects include the inhibition of the gastric jejunal and colonic motility [24–26]. Due to these properties, it is used alone or in conjunction with other adjuvant agents, in patients with soft esophageal impaction. Regarding the motility effect of Glucagon on the esophagus, a significant reduction in the mean resting pressure of the lower esophageal sphincter was noted with increased dosages of IV Glucagon (0.25 and 0.5 mg). The mean lower esophageal relaxation was significantly reduced after an IV dose of 0.25 mg of Glucagon. At higher doses (0.5 mg versus 1.0 mg), no further reduction in any lower esophageal sphincter functional parameters was observed. Also, there was no appreciable effect on the proximal amplitude of contraction and proximal or distal esophageal contraction [27]. Regarding the other segments of the esophageal body, significant reduction associated with IV Glucagon was seen with respect to the amplitude of contraction in the mid and distal esophagus, as well as diminished esophageal stripping, as shown using fluoroscopy [28]. These effects on the motility showed no or little effect on the relaxation of the smooth muscles containing the structures such as distal esophageal rings or strictures, when IV Glucagon was administered [29].

Dose and administration: intravenous 0.25 or 0.50 mg is used, and a latency period of 30 to 60 seconds is expected prior to its taking action on the smooth muscles of the oesophagus, with an action period lasting between 4 and 15 minutes, depending on the dosage. Contraindications include hypersensitivity to Glucagon and a history of pheochromocytoma or insulinoma. Side effects include nausea, vomiting, vague abdominal distress, diarrhea, skin rash, or dry mouth [30, 31].

### 5.3. Papaveretum

This is a preparation containing a mixture of the hydrochloride salts of opium alkaloids. Since 1993, papaveretum has been defined in the British Pharmacopoeia (BP) as a mixture of morphine hydrochloride, papaverine hydrochloride, and codeine hydrochloride. A single report showed the use of papaveretum in the management of esophageal impaction in a dose of (0.3 mg/Kg body weight) reported dislodgement of the food bolus within 12 hours in thirteen out of fifteen patients, which is attributed to increase in the tone of the smooth muscle of the esophagus, and papaveretum would calm the intense anxiety associated with the event [32]. 

### 5.4. Benzodiazepines

The muscle spasms associated with food bolus impaction were managed using IV diazepam 2.5–10 mg, according to weight and age, in a randomized study, supplemented by the concurrent administration of IV Glucagon if no response to IV diazepam was observed [33, 34].

### 5.5. Calcium Channel Blockers (CCB)

These chemical compounds used in the treatment of ischemic heart disease and systemic hypertension exert their effects by depletingtheintracellular calcium and modulating the smooth muscles, particularly the smooth muscles of the esophagus. Several studies done to investigate the effects of nifedipine on the manometric features on a normal oesophagus, chiefly the mean basal, amplitude, and lower esophageal sphincter (LES) pressures, showed a reduction in these parameters [35–37]. A report by Elson showed a successful esophageal disimpaction with the use of a 10 mg dose of sublingual liquid nifedipine [38]. When nifedipine was used to treat different dysmotility disorders, patients with diffuse esophageal spasm, achalasia, and nutcracker esophagus reportedly showed a significant decrease in the LES pressure and amplitude [36, 39, 40]. Verapamil is another CCB that has been reported to decrease the LES pressure when used in both oral and intravenous forms [41, 42]. However, the available evidence above supports that this group of medications relieves esophageal motility symptoms but currently no existent guidelines to suggest routine use of CCB in the acute management of esophageal impaction.

### 5.6. Nitrates

Isosorbide nitrates when used as 5 mg sublingual dose reportedly caused a significant drop in the mean basal LES pressure along with a significant decrease in the esophageal radionuclide test meal retention when compared with nifedipine 20 mg in patients with achalasia [37]. Nitrates have not yet been used to treat acute food impaction on a regular basis.

### 5.7. Papain

This powerful trypsin-like enzyme, capable of digestion, is derived from a tropical melon tree. It is commercially available as a household meat tenderizer. Several reports have claimed an effect on and against digesting an impacted food bolus. When used along with IV Glucagon, it facilitates the digestion of the food bolus impaction, particularly those of a meaty nature [43]. It is administered as 2.5% suspension of 2 tablespoons in 240 mL of water to be taken as 20 mL sips [44]. Alternatively an experimental study by Goldner and Danley showed that Adolph's Meat Tenderizer (AMT) solution has no inherent capacity to digest or to reduce the size of an impacted meat bolus and may, in fact, worsen the existing esophagitis when tested on animal model esophagus [45]. Lethal adverse events were noted to occur with its use, with significant transmural digestion of the esophageal wall itself and consequently fatal mediastinitis [43, 46].

### 5.8. Water

Water is normally given with Glucagon to facilitate dislodgement by virtue of gravity, besides assisting in liquefying the masticated food bolus. 

### 5.9. Effervescent Agents

E-Z gas was used in combination with IV Glucagon. It consists of sodium bicarbonate, citric acid, and simethicone and is dissolved in 30 mL of water along with 1 mg of IV Glucagon to induce gas formation and push the bolus downward [47]; another agent used as a gas-forming method is tartaric acid, followed immediately by sodium bicarbonate. Both can produce carbon dioxide in the oesophagus which helps push the food bolus into the stomach [48, 49]. Interestingly, when Coca-Cola was studied as a gas-forming agent and tested on a small group of patients with food bolus impaction, without the use of Glucagon, some patients experienced clearance of the impaction [49, 50].

## 6. Clinical Outcome and Prognosis

Patients with esophageal impactions have the potential of spontaneous dislodgement during observation period that may last for 24 hours, while others require more prompt response [51]. No predictable factor would guide the group of patients who benefit from expectant management. Delayed intervention may lead to further clinical distress or development of complications such as perforation.

### 6.1. Esophageal Perforation

It occurs when sharp pointed food items such as bone or soft food remain obstructing the esophagus for a duration exceeding 24 hours. Although it is uncommon (occurs in less than 1%), it requires major surgery. Perforation occurs as a result of prolonged food bolus exerting firm and constant pressure that results in ischemia-induced necrosis; this mechanism along with accumulated saliva that pools down would aggravate further pressure. Acute perforation manifests as retrosternal pain associated with shortness of breath, fever, and possible subcutaneous crepitus. Chest X-ray would show features of free air in the form of mediastinal widening, pneumomediastinum, pleural effusion, or hydropneumothorax. Prompt diagnosis and management are needed to extract the bolus surgically and repair the involved area of the esophagus [52, 53].

### 6.2. Recurrence of Esophageal Impaction

Recurrence of esophageal impaction is related to the underlying etiology, particularly if the patient was treated medically and if the condition was amenable to medical or endoscopic therapy. A series of patients in UK showed that hiatal hernia is the anomaly that is frequently noted in association with a recurrence of the impaction (odds ratio 4.77) [54].

## 7. Suggested Management Plan

**Figure 1:**
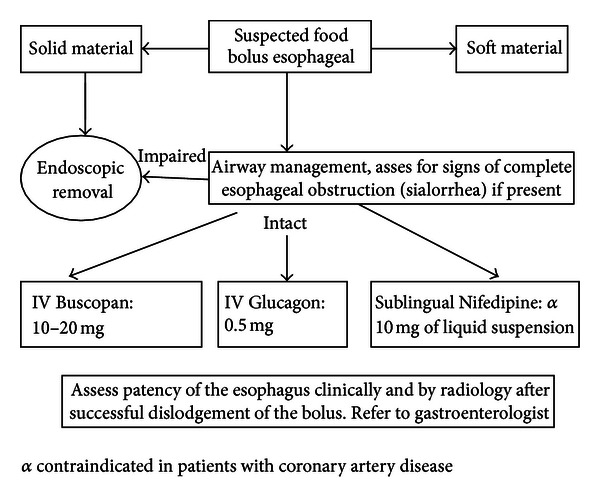
Suggested algorithm for management of suspected food bolus impaction.

For patients presenting with esophageal impaction symptoms, airway management is the first priority followed by focused history and clinical examination to reveal the presence of any possible known type of esophageal obstructing condition or if prior esophageal dilatation had been done. The knowledge of any kind of coexisting chronic medical illnesses is important to make the right choice of safe medical agent such as the presence of CNS and valvular or ischemic heart conditions. A history of any trace of allergy to Glucagon is important to avoid possible reactions when it is used. Monitoring of vital signs and signs of airway compromise is absolutely necessary during the patient's emergency visit (Figure 1). Caution should be exercised if papaveretum or benzodiazepines were chosen to be used in individuals or patients with impaired sensorium, elderly or those with underlying CNS condition because it may impair their capability to protect their upper airways. Communication with a skilled endoscopist capable of managing esophageal impaction is crucial to obtain a rapid relief if the medical options performed were not successful.

## 8. Conclusion

With the diversity in the types of treatments available for impacted food bolus, there is yet no proven superiority for any particular agent over another, based on randomized clinical trials [55]. However, with the variety of agents discussed and with the availability of appropriate evidence, medical treatment could be used with caution among the various treatment armamentariums currently available for treating patients with acute esophageal food impaction.

## Figures and Tables

**Box 1:**
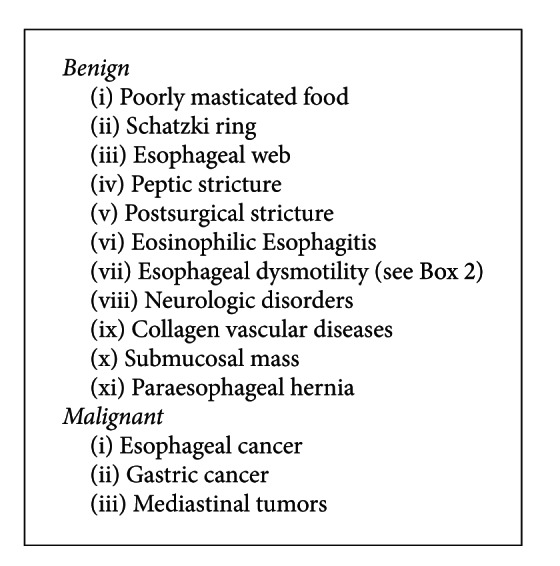
Causes of esophageal impaction.

**Box 2:**
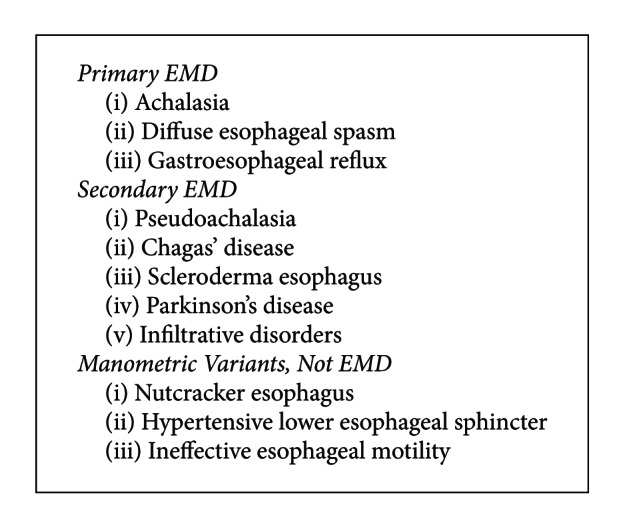
Esophageal Motility Disorders (EMD).
